# Influence of Phenotypes on Short-Term Outcomes in Hospitalized Heart Failure with Preserved Ejection Fraction—Insights from a North-Eastern Romanian Cohort

**DOI:** 10.3390/medsci14020167

**Published:** 2026-03-27

**Authors:** Victoria Mutruc, Mara Sânziana Sângeap, Cristina Bologa, Victorița Sorodoc, Ovidiu Rusalim Petriș, Oana Sîrbu, Bianca Codrina Morărașu, Luiza Elena Corneanu, Elisabeta Jaba, Laurențiu Șorodoc, Cătălina Lionte

**Affiliations:** 1 Faculty of Medicine, Grigore T. Popa University of Medicine and Pharmacy, 700115 Iasi, Romania; 2 Second Internal Medicine Department, Sfântul Spiridon Clinical County Emergency Hospital, 700111 Iasi, Romania; 3 Statistics Department, Alexandru Ioan Cuza University, 700506 Iasi, Romania

**Keywords:** cluster analysis, clinical phenotypes, comorbidities, HFpEF, real-world cohort, short-term outcomes

## Abstract

**Background/Objectives**: There are significant gaps in knowledge regarding the heterogeneity of heart failure (HF) phenotypes, particularly among patients with preserved left ventricular ejection fraction (HFpEF). Our aim was to identify phenotypes within the hospitalized North-Eastern Romanian HFpEF cohort and their impact on short-term outcomes. **Methods and Results**: We derived a cluster model from 924 patients with HFpEF hospitalized over an 18-month interval in the Internal Medicine II Department of the “Sf. Spiridon” Emergency Clinical County Hospital in Iași, Romania. The median age of the patients was 74 years [range 30–101], 59.8% were women, and the most frequent comorbidities were arterial hypertension (93.2%), valvular heart disease (68.7%), atherosclerotic cardiovascular disease (ASCVD, 64.6%) and atrial fibrillation (43%). Statistical analysis identified five distinct phenotypes: cluster 1 (21.6% of patients) consisted of normal-weight patients with valvular disease predominance; cluster 2 (18.2%) described a severe cardiometabolic phenotype; cluster 3 (19.6%) defined a young, hypertensive, and atherosclerotic phenotype; cluster 4 (21.26%) described a hypertensive–atrial fibrillation phenotype; and cluster 5 (18.9%) included elderly, hypertensive non-diabetic patients with severe vascular burden (ASCVD 100%). **Conclusions**: This study defines five distinct phenotypes within the HFpEF population in our region which differ in terms of clinical characteristics and heart failure pharmacological therapy. These results confirm the significant heterogeneity of HFpEF. The identified phenotypes were not associated with significant differences in composite short-term outcomes, including in-hospital mortality and 30-day rehospitalization for heart failure.

## 1. Introduction

Heart failure with preserved ejection fraction (HFpEF) is a complex syndrome, characterized by multiple systemic alterations and marked heterogeneity in pathophysiological mechanisms and clinical profiles [1]. HFpEF represents a growing proportion of HF cases worldwide; however, therapy remains challenging, and guidelines adherence suboptimal. Although numerous randomized clinical trials have evaluated different treatment strategies in this population, most have failed to demonstrate significant reductions in mortality and morbidity, with largely neutral results, a finding commonly attributed to the biological and clinical heterogeneity of the syndrome [2,3,4,5,6].

In this context, the recent literature suggests an approach based on homogeneous clinical phenotypes, identified using cluster analysis methods applied to routinely available clinical variables, with the potential to tailor therapeutic interventions to individual patient profiles [7,8,9]. Moreover, data on multimorbidity indicate that not only the presence of comorbidities, but also their specific combinations, significantly influence mortality risk in patients with heart failure [8]. These observations support the utility of identifying clinical phenotypes that account for patterns of comorbidities, rather than their number alone, to achieve more accurate outcome prediction.

Frequently reported HFpEF phenotypes include younger patients, with a relatively low comorbidity burden, described across cohorts with mean ages of approximately 55–60 years [7,10,11]. Other described phenotypes include AF and hypertension [7,12], elderly multimorbid phenotypes [8,9], and cardiometabolic phenotype characterized by obesity and DM [10,12,13]. These phenotypes differ substantially in terms of clinical characteristics, treatment patterns, and prognosis, supporting the role of clinical phenotyping in HFpEF. Beyond specific clinical phenotypes, multiple studies using cluster analysis and machine learning approaches have consistently demonstrated the marked heterogeneity of HFpEF populations and the prognostic relevance of data-driven phenotyping in real-world cohorts [14,15,16,17]. This phenotypic heterogeneity is increasingly interpreted within the broader framework of systemic cardio–renal–metabolic interactions, as highlighted by the cardiovascular–kidney–metabolic (CKM) syndrome, which integrates shared pathophysiological pathways across multiple organ systems in heart failure [18].

Cluster analysis is an unsupervised statistical method that enables the automatic grouping of individuals or variables based on similarities, without predefined group assignments. In the medical field, this technique has been used to identify patient subgroups with similar clinical features, comorbidity profiles, or biological outcomes. Although its routine clinical application is still evolving, cluster analysis has demonstrated potential in differentiating disease phenotypes and improving risk stratification [19].

Given the heterogeneity of HFpEF, the present study aimed to characterize a real-life cohort of hospitalized patients with HFpEF and to identify distinct clinical phenotypes using cluster analysis based on routinely available clinical variables. We further evaluated the distribution of comorbidities within these clusters, their relationship with short-term outcome, and markers of disease severity. Such an integrated approach may contribute to more precise risk stratification and support the individualization of treatment strategies.

## 2. Materials and Methods

**Design:** This was a retrospective, single-center study conducted at the Second Internal Medicine Department of the “Sf. Spiridon” Clinical County Emergency Hospital in Iași, Romania, and included 924 hospitalized patients with a documented LVEF ≥ 50% over an 18-month interval (Supplementary Figure S1). The study population was ethnically homogeneous, consisting of caucasian patients, reflecting the demographic structure of the North-Eastern Romanian region. Adult consecutive patients (≥18 years) with a primary or secondary diagnosis of heart failure with preserved ejection fraction (HFpEF) hospitalized in our center were included. Patients were identified retrospectively from hospital medical records. The diagnosis of HFpEF was made according to current guideline recommendations. The study reflects a real-world cohort. **Data sources:** Clinical data were obtained through retrospective review of hospital medical records. Information on patient demographics, clinical status, comorbidities, laboratory investigations, electrocardiographic and echocardiographic findings, and pharmacological treatment during hospitalization was collected. All data were entered into a dedicated database for analysis. Short-term outcomes, including in-hospital mortality and 30-day rehospitalization, were assessed using the INFO World Health Information System (HIS)**,** which is widely used in hospitals across Romania. **Statistical analysis:** Each variable distribution was presented as mean ± SD, median with interquartile range, or frequency. Student’s *t* test or Mann–Whitney U test for numerical variables, as well as the Chi-square test and Cochrane’s statistic for categorical variables, were used to compare if significant differences exist between survivors and non-survivors. A *p* value < 0.05 was considered statistically significant.

For the identification of clinical phenotypes, the TwoStep Cluster analysis in SPSS version 23 was used. The variables included in the cluster analysis were selected based on their ability to discriminate distinct clinical profiles, using the predictor importance generated by the model. The quality of the clustering model was evaluated using the Silhouette measure of cohesion and separation, which had a value of 0.6, indicating a good separation between clusters. Adding continuous variables significantly correlated with the outcome (i.e., C-reactive protein, D-dimers) did not improve the quality of the clustering model. The optimal number of clusters was additionally explored using the Elbow method [20]. Short-term prognosis was evaluated during hospitalization and up to 30 days of follow-up. In-hospital mortality and 30-day rehospitalization were analyzed across HFpEF clusters. A composite short-term outcome was defined as in-hospital death and 30-day rehospitalization. Cumulative incidence curves were constructed to assess the timing of event occurrence. Detailed time-to-event analyses were performed using Stata software, version 17. Statistical analysis was performed using IBM SPSS Statistics software, version 23, and graphical representations were created using PowerPoint and BioRender.com.

The study was conducted in accordance with the principles outlined in the Declaration of Helsinki. The study was approved by the local research ethics committee of the Grigore T. Popa University of Medicine and Pharmacy, Iași, Romania (approval no. 537, date of approval: 13 February 2025), and by the ethics committee of the “Sf. Spiridon” Clinical County Emergency Hospital (approval no. 15, date of approval: 22 January 2025).

## 3. Results

A total of 924 patients with a diagnosis of HFpEF were included, of whom 59.8% were women (553 patients). The median age of the patients was 74 years [67–81.0], reflecting a predominantly elderly population. The prevalence of comorbidities, the distribution of the main laboratory parameters, and pharmacological treatments are presented in Supplementary Table S1.

Most patients were admitted for mild or moderate HF symptoms, with 82.7% classified as NYHA class I–II and 17.3% as NYHA class III–IV. Regarding cardiovascular comorbidities, hypertension was the most frequent (93.2%), followed by valvular heart disease (68.7%); ASCVD was documented in 64.6% of patients (of whom 51.7% had ischemic heart disease, 6.3% peripheral artery disease and cerebrovascular disease 6.6%) and atrial fibrillation in 43%. Among non-cardiovascular comorbidities, diabetes mellitus/prediabetes was present in 44.8% of patients, while overweight/obesity (BMI ≥ 25 kg/m^2^) was observed in 78.9%. Renal function was moderately impaired in 25.6% of patients (eGFR 30–60 mL/min/1.73 m^2^) and severely reduced in 5%. The presence of chronic kidney disease did not correlate significantly with the composite outcome, although it correlates significantly with death during hospitalization (5.4% vs. 1%, *p* < 0.001). Antihypertensive therapies (ACE inhibitors/ARBs/ARNIs and beta-blockers) and statins were prescribed in a high proportion of patients, reflecting the high prevalence of hypertension and ASCVD. Diuretics were also frequently administered, consistent with the need for congestion control. The use of anticoagulants corresponded to the prevalence of atrial fibrillation, and SGLT2 inhibitors were prescribed in 44.2% of patients, a suboptimal percentage given the guidelines recommendations [21], representing the practical challenges in real life, since reimbursement often requires strict adherence to therapeutic protocols (e.g., NT-proBNP levels > 300 pg/mL in sinus rhythm and >600 pg/mL for patients with atrial fibrillation). The timing of SGLT2 inhibitor initiation was not specifically analyzed in our study. It is possible that delayed introduction of therapy may have influenced the absence of a detectable short-term mortality benefit. Future studies evaluating time-to-treatment initiation may provide additional insights.

In this retrospective study, the overall mortality rate was 2.4%. Non-survivors were older (*p* < 0.014) and more frequently presented with advanced symptoms, with 18 of 22 deaths (81.8%) occurring in patients classified as NYHA class III–IV (*p* < 0.001). Non-survivors also had significantly higher NT-proBNP levels (6393 [2127–15,357] vs. 637 [188–2546] pg/mL in survivors; *p* < 0.001), suggesting a strong association between symptom severity, cardiac burden, and in-hospital mortality. The biological profile of non-survivors showed significantly higher levels of hs-cTnI, D-dimers, and CRP, together with more severe renal impairment (*p* < 0.001). Full details are presented in Table 1.

### 3.1. Cluster Analysis

**Figure 1:**
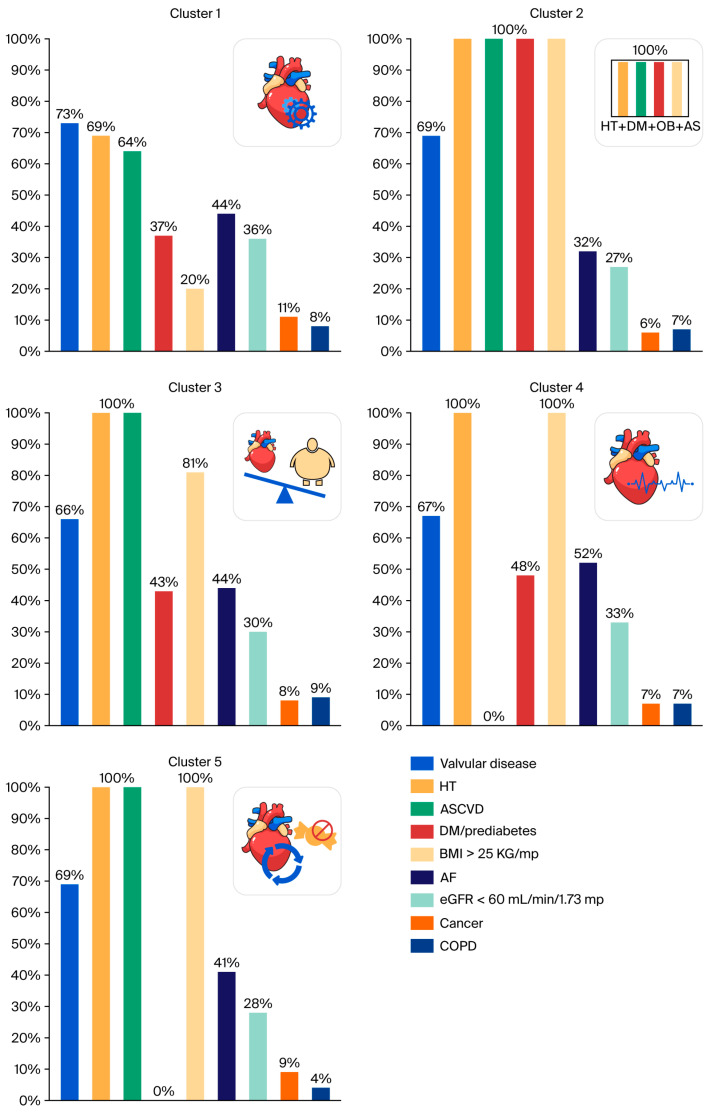
Patient comorbidity profiles within clusters. Abbreviations: Overweight/Obesity: body mass index ≥ 25 kg/m^2^. COPD: chronic obstructive pulmonary disease, ASCVD: Atherosclerotic Cardiovascular Disease.

Cluster analysis based on the following variables, age ≥ 65 years, DM/prediabetes, ASCVD, hypertension, and BMI ≥ 25 kg/m^2^, identified five distinct phenotypes in HFpEF: cluster 1: non-metabolic phenotype with valvular disease predominance; cluster 2: cardiometabolic phenotype; cluster 3: young vascular phenotype; cluster 4: hypertensive–atrial fibrillation phenotype; cluster 5: atherosclerotic non-diabetic HFpEF phenotype (Figure 1).

The main characteristics according to cluster classification are presented in Table 2. All variables used to identify the cluster showed significant differences between the clusters (*p* < 0.001).

### 3.2. Baseline Characteristics and Clinical Profile

Cluster 1 included 200 patients (21.6%) and describes a non-metabolic HFpEF phenotype with valvular disease predominance (Figure 1). It is the only cluster in which most patients were of normal weight, with 80.5% having a BMI < 25 kg/m^2^, a feature that clearly differentiates it from all other clusters, in which overweight/obesity was universal. This phenotype comprised predominantly elderly patients (95% aged ≥65 years), mainly females; the median age was 76 years, comparable to that observed in clusters 2, 4, and 5, indicating that age alone did not represent a discriminative feature of this phenotype. It was characterized by a marked predominance of valvular heart disease, together with a moderate burden of hypertension and ASCVD, without the severe cardiometabolic load observed in other clusters. A distinctive feature was the highest median NT-proBNP level (1015 pg/mL) among all clusters. This finding may be interpreted in the context of a non-obese status [22,23].

Cluster 2 included 168 patients (18.2%) and represents a cardiometabolic HFpEF phenotype (Figure 1). This cluster was characterized by advanced age and the universal presence of obesity, hypertension, DM/prediabetes, and ASCVD (100% for all), defining the most severe cardiometabolic profile within the cohort. This subgroup showed the highest use of RAAS inhibitors, CCB, statins, and SGLT2 inhibitors, supporting the presence of an HFpEF phenotype with advanced cardiometabolic burden.

Cluster 3 included 181 patients (19.6%) and represents a young vascular HFpEF phenotype (Figure 1). Patients were significantly younger (median age 60 years), all had hypertension, and exhibited a high prevalence of ASCVD (70.2%), indicating early-onset atherosclerotic disease. The metabolic profile was moderate, with DM/prediabetes present in 43% of patients.

Cluster 4 included 200 patients (21.6%) and may be classified as a hypertensive–atrial fibrillation HFpEF phenotype (Figure 1). Patients were elderly (median age 75 years), predominantly female, with universal hypertension and overweight/obesity. AF was most prevalent in this group (52%), reflecting age-related cardiac remodeling. This cluster was distinguished by the complete absence of ASCVD. The median NT-proBNP value of 914.5 pg/mL reflects atrial dilatation and stretch associated with AF [24].

Cluster 5 included 175 patients (18.9%) and represents an atherosclerotic HFpEF phenotype (Figure 1), characterized by the coexistence of obesity, hypertension, and ASCVD in all patients. This phenotype showed a vascular profile similar to that of the cardiometabolic cluster but was clearly differentiated by the complete absence of DM/prediabetes. Thus, cluster 5 delineates a subgroup of HFpEF patients in whom atherosclerotic vascular involvement occurs independently of glycemic dysfunction. In clusters 2, 4, and 5, in which BMI ≥ 25 kg/m^2^ was present in 100% of patients, the relatively lower NT-proBNP levels may reflect an obesity-associated natriuretic peptide deficiency syndrome in HFpEF, characterized by an inappropriately blunted natriuretic peptide response relative to hemodynamic load and clinical severity, rather than true disease absence [22,23].

The clusters associated with highest number of fatalities were 1, 4, 5, and the clusters associated with the highest number of patients in need of 30 days rehospitalization for HF were 1 and 4. However, despite the fact that identified phenotypes displayed clear clinical differences, these were not accompanied by significant differences in short-term prognosis, including in-hospital mortality or 30-day rehospitalization rates (Table 2).

### 3.3. Prognosis

For the overall cohort, short-term prognosis was evaluated during hospitalization and up to 30 days of follow-up. We assessed the association between HFpEF clusters and in-hospital mortality, as well as 30-day rehospitalization for HF, as the individual components of the composite short-term outcome (Supplementary Figure S2). We performed a cause-specific Cox proportional hazard model censoring for in-hospital mortality and HF hospitalization events, respectively. Results are presented as hazard ratios (HR) with 95% confidence intervals (95% CI) and visualized with cumulative incidence curves. We adjusted the analyses for age and sex. Although the overall frequency of in-hospital mortality and 30-day rehospitalization did not differ significantly between clusters, the cumulative incidence curves revealed differences in the temporal dynamics of event occurrence (Supplementary Figure S2).

In the analysis of in-hospital mortality (Supplementary Figure S2a), the curves suggested an earlier occurrence of deaths in clusters 4, 5, and 1, compared with clusters 2 and 3, which showed a more delayed distribution of events during hospitalization.

In the analysis of the composite short-term outcome (Supplementary Figure S2b), cumulative incidence curves suggested differences in the timing of events between clusters, with a tendency toward earlier event occurrence in clusters 1 and 4, whereas clusters 2, 3, and 5 exhibited a more favorable short-term course. Detailed statistical analyses underlying the cumulative incidence curves are provided in the Supplementary Table S2.

In summary of the findings, these results highlight the presence of differences in the temporal dynamics of short-term prognosis across HFpEF clusters, with respect to both in-hospital mortality and 30-day rehospitalization, even in the absence of significant differences in the overall frequency of events.

## 4. Discussion

This study had as its primary objective the phenotypic analysis of HFpEF in an Eastern Romania cohort of patients hospitalized for HF in relation with short-term outcomes. We observed that patients were predominantly elderly, mostly female, with a high prevalence of hypertension and metabolic comorbidities. Overall mortality was low (2.4%). Deceased patients were older, more frequently presented with advanced NYHA functional classes, markedly elevated NT-proBNP levels, and significantly impaired renal function. These findings are consistent with the data reported by Yang et al. [25], who showed that mortality in HFpEF is strongly influenced by combinations of comorbidities, particularly cardio-renal and cardiometabolic ones. Moreover, the authors showed that reduced renal function and elevated NT-proBNP represent consistent and independent predictors of adverse prognosis, which is also observed in our cohort. Interestingly, obesity was less frequent among deceased patients (0.9%), a phenomenon that may suggest the presence of the “obesity paradox”. In a recent analysis of patients with HFpEF, a higher body mass index was associated with lower mortality [26].

Phenotyping of HFpEF patients using machine learning techniques has been extensively explored in the recent literature, with numerous international studies identifying between three and six distinct clusters. A comparative synthesis of these cluster analyses is presented in Table 3.

Cluster 1 included elderly patients, mostly of normal weight (80.5%), with a predominance of valvular heart disease, a moderate prevalence of hypertension and ASCVD, and the lowest prevalence of DM/prediabetes. This pattern is not observed in international phenotyping studies [9,10,11,12,13,14,15,16], where elderly subgroups are predominantly cardiometabolic, multimorbid, or characterized by a predominance of AF. Therefore, cluster 1 appears to represent a distinct phenotype of our cohort, characterized by advanced age and low metabolic risk. This cluster exhibited the highest prevalence of valvular heart disease (73%). Valvular heart disease (VHD) is common in HFpEF. Functional tricuspid regurgitation (TR) affects 20–50%, and functional mitral regurgitation (MR) affects up to 20% of all patients with HFpEF. TR has been identified as an independent predictor of adverse outcomes and mortality [27]. There is still lack of deep understanding of the pathogenic mechanism of VHD-related HF subtypes. The mechanism of VHD-related HF involves not only mechanical damage to the valve itself but also valve lesions caused by myocardial ischemia or mitochondrial dysfunction. The interactions between them will lead to the occurrence and development of VHD-related HF subtypes. Valvular disease increases left atrial (LA) pressure, leading to atrial remodeling and dysfunction, which drives diastolic dysfunction. Functional MR is common, associated with LA distension, and often acts as a marker of advanced, irreversible myocardial dysfunction. Significant TR is common in this group, serving as a powerful independent predictor of adverse outcomes [28]. Chronic pressure and/or volume overload affecting the left heart may lead to progressive atrial and ventricular remodeling, increased myocardial wall stress, and impaired ventricular compliance. The resulting structural and hemodynamic vulnerability may promote early clinical destabilization and earlier event occurrence, even in the absence of a severe metabolic risk profile compared with other clusters. Due to the combination of different valvular lesions and the sequence of valvular damages, the patient’s condition is more complicated and represents a dilemma when physicians decide on a treatment plan [28].

Data from the Chinese Cardiovascular Association Database-Heart Failure Center Registry showed, after 1-year follow-up, that valvular/rhythm-related phenotypes were associated with increased heart failure rehospitalization, while extracardiac disease-related HFpEF was linked to higher cardiovascular mortality [29]. In patients with acute decompensated HFpEF and with over 5 years of follow-up, nearly two-thirds of patients died, half from CV and the other half from non-CV causes. CAD and tricuspid regurgitation were associated with CV death [30].

The unfavorable short-term outcomes observed in cluster 1 may also be explained by frailty, a highly prevalent syndrome among elderly patients hospitalized for HF, which is associated with poor prognosis and a 1.5–2-fold increased risk of all-cause mortality and hospitalization [31].

The cardiometabolic cluster 2 is comparable to phenotypes reproduced in other cohorts. Uijl et al. described a cluster 4 (15% of the HFpEF population) dominated by obesity, DM and hypertension [12], while Vicent et al. identified a cluster B, composed predominantly of women with diabetes mellitus and multiple comorbidities, associated with the shortest median survival (90 days) and a 1-year mortality of approximately 33% [14]. Similar results have also been reported in other international analyses [9,10,13,15,16,17]. The age and severity of vascular disease observed in younger patients from cluster 3 represent a notable particularity of our cohort (median age 60 years, 100% hypertensive, with ASCVD in 69% and intensive lipid-lowering therapy in 41%). This profile is rarely observed in other HFpEF populations, where younger patients are often considered “low risk” [9,10] or exhibit a non-atherosclerotic phenotype [16,17]. Thus, the young vascular HFpEF’’ cluster appears to constitute a distinct and rarely reported phenotype, suggesting a particular expression of the disease in this Eastern Romanian cohort. Cluster 4 in our cohort, characterized by the complete absence of ASCVD and a high prevalence of hypertension and atrial fibrillation, most closely resembles the “atrial fibrillation–hypertension” phenotypes described in other studies [10,12,13,14], but with a higher proportion of overweight patients and those with diabetes mellitus/prediabetes. Through its overall low vascular risk profile, it also shows similarities with the low-risk phenotypes reported in other studies [17]. By contrast, cluster 5 included elderly, obese, hypertensive patients with ASCVD, but with complete absence of diabetes mellitus/prediabetes. This phenotype represents a rarely reported association in the international literature [10,11,12,13,14,15,16,17], where phenotypes with severe ASCVD are usually classified within cardiometabolic phenotypes.

Phenotypic classification in HFpEF has a well-established prognostic value over the medium and long term [30,31]. Our results suggest that phenotyping may also be relevant for early prognosis, even though differences between phenotypes did not reach statistical significance for in-hospital mortality or 30-day rehospitalization.

The lack of statistical significance should be interpreted in the context of a low number of events and a short follow-up duration. The novelty of our study lies in highlighting that HFpEF phenotyping can capture early differences in clinical vulnerability, as reflected by distinct early clinical trajectories (Supplementary Figure S2a,b), which may become more apparent with longer follow-up.

Cumulative incidence analyses indicated the presence of differences in early clinical trajectories between phenotypes. This observation is consistent with the findings reported by Miró and colleagues [13], who showed that, in cohorts of hospitalized HFpEF patients, clinical phenotypes may be associated with distinct early evolution patterns, even when the overall frequency of events does not differ significantly between groups.

By contrast, studies with longer follow-up periods have demonstrated a clearer prognostic relevance of HFpEF phenotyping, with differences between clusters becoming more evident over time [10,11,12,14]. These data support the hypothesis that the prognostic impact of HFpEF phenotypes is time-dependent, being more apparent over the medium to long term than during the acute or immediate post-hospitalization phase.

## 5. Limitations

Our study has several limitations that should be considered when interpreting the results. First, the retrospective and single-center design limits the possibility of establishing causal relationships and may be associated with selection bias. In addition, the cohort included exclusively hospitalized patients with HFpEF, which may not fully reflect outpatient populations and limits the generalizability of the results. We did not use a validated HF specific frailty assessment tool, as such a tool does not exist currently. Prognostic evaluation was limited to in-hospital mortality and 30-day rehospitalization. The short follow-up duration, as well as the low event rate, reduces the ability to identify significant prognostic differences between the identified phenotypes. The relatively low number of short-term events may affect the precision of statistical estimates and may explain the wide confidence intervals observed in the Cox models. In this context, the possibility of model overfitting cannot be excluded, and the results should therefore be interpreted with caution. Although the variables were selected based on predictor importance, the structure of some clusters remains influenced by the set of variables included in the model, particularly in the case of phenotypes defined by the presence or absence of diabetes mellitus/prediabetes and atherosclerotic cardiovascular disease. The clustering model was based on routinely available clinical variables, predominantly dichotomized for analytical purposes, which may influence cluster structure. In addition, external validation of the clusters in an independent cohort was not yet performed, which may limit the robustness and reproducibility of the identified phenotypes and require confirmation in independent cohorts. We did not assess the influence of variables reflecting endothelial stress, other than high-sensitivity CRP, since the other biomarkers (i.e., adrenomedullin, P-selectin) or non-invasive methods, such as flow-mediated dilation, are not routinely used in clinical practice [32,33].

Information regarding the duration of heart failure prior to index hospitalization was not systematically available in the retrospective dataset used. This variable could have provided additional insights into the chronic disease trajectory and may contribute to a more nuanced characterization of phenotypes in future prospective studies.

As a result, prospective, multicenter studies with longer follow-up periods and external validation are required to confirm the robustness and clinical relevance of the identified phenotypes in this North-Eastern Romanian cohort

## 6. Conclusions

In this real-world cohort of hospitalized patients with HFpEF, we identified five distinct clinical phenotypes using simple and readily available variables, highlighting substantial heterogeneity characterized by different comorbidity profiles. We observed a tendency toward earlier event occurrence in clusters 1 and 4, whereas clusters 2, 3, and 5 exhibited a more favorable short-term course. These subgroups may facilitate more accurate patient stratification and represent a step towards personalized management in HFpEF. There is a need for identifying barriers in guidelines implementation in the HFpEF European population.

## Figures and Tables

**Table 1:** Characteristics of non-survivors and survivors in patients with HFpEF.

Variable	Non-Survivors N = 22 (2.4%)	Survivors N = 902 (97.6%)	*p* Value
**Demographics**			
Female sex, n (%)	10 (45.5)	543 (60.2)	0.189
Age, median [IQR]	78.5 [74–86.2]	74 [67–81]	0.014
**HF measurements**			
H2FPEF score	4 [3–5]	4 [3–5]	0.495
NYHA class I/II, n (%)	4 (18.2)	760 (84.3)	0.001
NYHA class III/IV, n (%)	18 (81.8)	142 (15.7)	0.001
NT-proBNP (pg/mL), median [IQR]	6393 [2127.2–15,357.0]	637 [188.5–2456]	<0.001
**Clinical measurements**			
SBP (mmHg), median [IQR]	135.0 [113.0–152.5]	139.0 [125.0–154.0]	0.336
HR, bpm, median [IQR]	83 [75–100]	76 [69–88]	0.002
SpO2,%, median [IQR]	95 [92–98]	97 [95–98]	0.009
BMI (kg/m^2^), n (%)			
<25	5 (22.7)	190 (21.1)	
≥25	17 (77.3)	712 (78.9)	0.795
**Comorbidities, n (%)**			
*Cardiovascular*			
Ischemic heart disease	5 (22.7)	395 (43.8)	0.052
Atrial fibrillation	13 (59.1)	384 (42.6)	0.132
Hypertension	19 (86.4)	84 (93.3)	0.185
Valvular disease	17 (77.3)	618 (68.5)	0.488
ASCVD	13 (59.1)	584 (64.7)	0.653
Implantable devices *, n (%)	2 (9.1)	29 (3.2)	0.166
*Noncardiovascular*			
COPD	2 (9.1)	61 (6.8)	0.657
Sleep apnea	2 (9.1)	45 (5.0)	0.309
Diabetes/Prediabetes	8 (36.4)	406 (45.0)	0.517
CKD/eGFR (mL/min/1.73 m^2^), n (%)			
≥60	6 (27.3)	624 (70.0)	
30–60	9 (40.9)	228 (25.6)	0.001
<30	7 (31.8)	39 (4.4)	
Malignant cancer	1 (4.8)	72 (8.1)	1.000
Iron deficiency, n (%)	9 (42.9)	356 (42.8)	1.000
**Laboratory investigations**			
*Biomarkers*			
hscTnI (ng/L), median [IQR]	15.7 [11.5–19.9]	4.35 [1.85–12.2]	0.050
D-dimers (mcg/mL), median [IQR]	3.8 [1.8–5.2]	1.02 [0.4–2.0]	<0.001
CRP (mg/dL), median [IQR]	6.3 [0.8–14.7]	0.3 [0.1–1.2]	<0.001
*Hematological tests*			
Hb (g/dL), median [IQR]	12.1 [9.9–13.9]	13 [11.5–14.4]	0.097
Leukocytes (mcgL)	9110 [6170–14,380]	7300 [5950–9250]	0.068
*Biochemical tests*			
eGFR (mL/min/1.73 m^2^), median [IQR]	43.3 [23.2–71.0]	77.0 [56.2–91.0]	<0.001
Blood glucose (mg/dL), median [IQR]	132 [110.2–195.7]	105.0 [93.0–125.0]	0.004
LDL-cholesterol (mg/dL), median [IQR]	101 [67.2–131.0]	92.0 [66.0–127.0]	0.870
Triglycerides (mg/dL), median [IQR]	82.0 [57.5–118.5]	93.0 [69.0–126.0]	0.380
Uric acid (mg/dL), median [IQR]	7.2 [5.1–11.0]	5.8 [4.8–7.1]	0.088
Ferritin (ng/mL), median [IQR]	274.0 [44.0–500.0]	89.0 [46.0–168.0]	0.070
*EKG (%)*			
¯ Normal EKG	5 (22.7)	471 (52.3)	
¯ Ischemia or myocardial infarction	4 (18.2)	73 (8.1)	0.680
¯ Tachyarrhythmias or bradyarrhythmias	2 (9.1)	53 (5.9)	
¯ AF or AFL	10 (45.5)	287 (31.9)	
¯ Pacemaker rhythm EKG	1 (4.5)	16 (1.8)	
*Echocardiography*			
LVEDD, mean ± SD	57.2 ± 8.8	47.3 ± 7.4	0.012
LVESD, mean ± SD	38.7 ± 8.6	32.9 ± 7.6	0.132
LVEF, 50–55%	15 (68.2)	617 (68.4)	1.000
LVEF, 56–64%	6 (27.3)	233 (25.8)	0.810
LVEF, ≥65%	1 (4.5)	49 (5.4)	1.00
**Medication and other interventions, n (%)**			
Diuretic	17 (77.3)	556 (61.6)	0.182
ACE inhibitor/ ARB/ARNI	9 (40.9)	687 (76.2)	<0.001
Beta-blocker	12 (54.5)	696 (77.2)	0.020
CCB	10 (45.5)	450 (49.9)	0.830
MRA	7 (31.8)	314 (34.8)	1.000
Antiaritmics	4 (18.2)	81 (9.0)	0.136
Statin	11 (50.0)	793 (87.9)	<0.001
Antiplatelet	2 (9.1)	245 (27.1)	0.058
Anticoagulant	16 (72.7)	400 (44.3)	<0.001
iSGLT2	9 (40.9)	399 (44.2)	0.830
Nitrates	2 (9.1)	123 (13.6)	0.756

* Implantable cardioverter-defibrillator, cardiac resynchronization therapy or pacemaker. Data are presented as mean ± SD, median (25th to 75th percentile) for continuous measures, and n (%) for categorical measures. Abbreviations: IQR: interquartile range, SD: standard deviation, NYHA: New York Heart Association, NT-proBNP: N-terminal pro-B-type natriuretic peptide, SBP: systolic blood pressure; HR: heart rate; BMI: body mass index, PAD: Peripheral Arterial Disease, ASCVD: Atherosclerotic Cardiovascular Disease, COPD: chronic obstructive pulmonary disease, CKD: chronic kidney disease, eGFR: estimated glomerular filtration rate, hscTnI: high-sensitivity cardiac Troponin I, Hb: Hemoglobin, AF: Atrial Fibrillation, AFL: Atrial Flutter, LVEDD: Left Ventricular End-Diastolic Diameter, LVESD: Left Ventricular End-Systolic Diameter, LVEF: left ventricular ejection fraction, ACE: angiotensin-converting enzyme, ARB: angiotensin receptor blocker, ARNI: Angiotensin-converting enzyme inhibitors, CCB: dihydropyridine calcium-channel blocker, iSGLT2: Sodium-Glucose Co-Transporter 2 inhibitor.

**Table 2:** Basal demographic, clinical characteristics, and laboratory parameters of the 5 clusters.

	Cluster 1	Cluster 2	Cluster 3	Cluster 4	Cluster 5	*p*-Value
Patients, n (%)	n = 200 (21.6)	n = 168 (18.2)	n = 181 (19.6)	n = 200 (21.26)	n = 175 (18.9)	
Age (years), median [IQR]	76 [71–82]	76 [72–83]	60 [54.5–63.0]	75 [72.0–82.7]	76 [72–83]	<0.001
Female sex, *n* (%) *	121 (60.5)	101 (60.1)	114 (64)	122 (61)	95 (54.3)	0.534
**HF measurements**						
H2FPEF score	3 [2–4]	4 [3–5]	4 [3–5]	4 [3–5]	4 [3–4]	<0.001
NYHA class I/II, n (%) *	150 (75.0)	147 (87.5)	152 (84.0)	161 (80.5)	154 (88.0)	0.004
NYHA class III/IV, *n* (%) *	50 (25.0)	21 (12.5)	29 (16.0)	39 (19.5)	21 (12.0)	0.004
NT-proBNP (pg/mL), median [IQR]	1015.0 [283.5–4310.6]	419.0 [146.5–1554.5]	682.1 [161.0–2121.0]	914.5 [205.0–3032.8]	436.0 [183.0–1994.0]	<0.001
**Clinical measurements**						
SBP (mmHg), median [IQR]	130 [118–149]	141 [130–157.5]	141 [125–160]	140 [125–153]	139 [125–152]	<0.001
HR, bpm, median [IQR]	79 [70–90]	75 [65–88]	78 [68–90]	78.5 [70.2–90]	75 [66–83]	0.015
BMI (kg/m^2^), n (%) *						
<25	161 (80.5)	0 (0.0)	34 (18.8)	0 (0.0)	0 (0.0)	<0.001
≥25	39 (19.5)	168 (100.0)	147 (81.2)	200 (100.0)	175 (100.0)	<0.001
Comorbidities, n (%) *						
*Cardiovascular*						
Atrial fibrillation	88 (44.0)	54 (32.1)	79 (43.6)	104 (52.0)	72 (41.1)	0.005
Hypertension	137 (68.5)	168 (100.0)	181 (100.0)	200 (100.0)	175 (100.0)	<0.001
Valvular disease	146 (73.0)	116 (69.0)	120 (66.3)	133 (66.5)	120 (68.6)	0.615
ASCVD	127 (63.5)	168 (100.0)	127 (70.2)	0 (0.0)	175 (100.0)	<0.001
Implantable devices **, n (%) *	7 (3.5)	7 (4.2)	8 (4.4)	4 (2.0)	5 (2.9)	0.689
*Noncardiovascular*						
COPD	16 (8.0)	11 (6.5)	16 (8.8)	13 (6.5)	7 (4.0)	0.428
Sleep apnea	2 (1.0)	10 (6.0)	14 (7.7)	16 (8.0)	5 (2.9)	0.004
DM/prediabetes	73 (36.5)	168 (100.0)	78 (43.1)	95 (47.5)	0 (0.0)	<0.001
CKDeGFR (mL/min/1.73 m^2^)						0.368
≥60	129 (64.5)	121 (72.9)	124 (69.7)	131 (67.2)	125 (71.8)	
30–60	63 (31.5)	35 (21.1)	47 (26.4)	50 (25.6)	42 (24.1)	
<30	8 (4.0)	10 (6.0)	7 (3.9)	14 (7.2)	7 (4.0)	
Malignant cancer	21 (10.6)	10 (6.0)	14 (7.8)	13 (6.7)	15 (8.7)	0.516
Iron deficiency	74 (40.2)	68 (42.0)	65 (39.6)	99 (53.8)	59 (37.1)	0.014
**Laboratory investigations**						
hscTnI (ng/L), median [IQR]	6.4 [2.9–19.4]	5.1 [1.6–15.4]	3.8 [1.6–12.4]	5.8 [1.8–20.0]	3.9 [1.1–13.7]	0.374
Hb (g/dL), median [IQR]	12.7 [11.2–14.3]	12.9 [11.6–14.0]	12.9 [10.8–14.1]	12.2 [10.4–13.9]	13.0 [11.9–14.8]	0.100
CRP (mg/dL), median [IQR]	0.3 [0.1–2.0]	0.4 [0.2–1.3]	0.5 [0.1–2.0]	0.8 [0.1–1.8]	0.6 [0.1–1.9]	0.364
Ferritin (ng/mL), median [IQR]	110.5 [47.7–277.5]	111.0 [47.2–201.7]	92.0 [53.0–181.5]	88.0 [41.7–138.5]	109.0 [53.0–185.0]	0.194
**Medication and other interventions, n (%)** *						
Diuretic	111 (55.5)	111 (66.1)	113 (62.4)	137 (68.5)	101 (57.7)	0.045
ACE inhibitor/ ARB/ARNI	121 (60.5)	139 (82.7)	145 (80.1)	154 (77.0)	138 (78.9)	<0.001
Beta-blocker	147 (73.5)	138 (82.1)	145 (80.1)	147 (73.5)	131 (74.9)	0.160
CCB	80 (40.0)	104 (61.9)	88 (48.6)	94 (47.0)	94 (53.7)	<0.001
MRA	73 (36.5)	61 (36.3)	56 (30.9)	73 (36.5)	58 (33.1)	0.721
Antiaritmics	18 (9.0)	12 (7.1)	13 (7.2)	20 (10.0)	22 (12.6)	0.370
Statins	154 (77.0)	159 (94.6)	162 (89.5)	172 (86.0)	157 (89.7)	<0.001
Antiplatelet	49 (24.5)	76 (45.3)	53 (29.3)	11 (5.5)	58 (33.1)	<0.001
Anticoagulant	96 (48)	53 (31.6)	87 (48.0)	105 (52.5)	76 (43.5)	0.038
iSGLT2	97 (48.5)	93 (55.4)	70 (38.7)	81 (40.5)	67 (38.3)	0.003
Nitrates	20 (10.0)	29 (17.3)	28 (15.5)	12 (6.0)	36 (20.6)	<0.001
Outcome, n (%) *						
In-hospital death	6 (3.0)	2 (1.2)	4 (2.2)	5 (2.5)	5 (2.9)	0.816
30 days -rehospitalization	32 (16.0)	20 (11.9)	25 (13.8)	33 (16.5)	16 (9.1)	0.217

* Percentage within cluster. ** Implantable cardioverter-defibrillator, cardiac resynchronization therapy or pacemaker.

**Table 3:** Phenotypic clusters in context of previous studies.

Author, Year	Data Set and Type/No. Patients	Method Clustering	Cluster 1	Cluster 2	Cluster 3	Cluster 4	Cluster 5	Cluster 6
Shah, 2015 [10]	EHR/397	Hierarchical clustering	Younger and low BNP	Obesity, DM and HT	Oldest with CKD and AF			
Kao, 2015 [17]	Trial/4113	Latent class analysis	Younger men with higher alcohol use	Younger women with anemia	Obesity, DM and CAD	Women with DM, obesity and CKD	Men with AF and CAD	Older, female with HT and AF
Cohen, 2020 [16]	Trial/3442	Latent class analysis	Younger, smoking	Older age, women and CKD and AF	Obesity, DM and CKD			
Hedman, 2020 [11]	Cohort/320	Model based clustering	Younger with HT, CAD and DM	Older with COPD, CKD and AF	Younger, male and obesity	Male, HT and AF	Older, female with HT and CAD	Older, female with HT and AF
Uijl, 2020 [12]	Registry/6909	Latent class analysis	Young, low comorbidity burden	AF and HT without DM	Oldest with many comorbidities, HT and AF	Obese, diabetic and HT, CAD	Older, with HT and AF	
Miró, 2025 [13]	Cohort/ 320	Model-based clustering	Older men, high COPD, CKD > 40%, HT > 90%	Younger, DM, dyslipidemia, HT and obesity	Oldest women, high HT, moderate CKD	Younger women, low comorbidity (valvular disease 50%, HT 55%, very low DM (16%)		
Vicent, 2025 [14]	Registry/1000	Two-step cluster	Women, low comorbidity burden	Women, high DM, CKD obesity, HT, dyslipidemia	Men, smokers, COPD, CAD, HT, CKD			
Mutruc, 2026	Cohort/924	Two-step cluster	Oldest with many comorbidities	Obesity, DM, ASCVD	Younger with ASCVD, HT and obesity	HT and AF	HT, ASCVD, obesity, no DM	

CKD, chronic kidney disease; HT, hypertension; AF, atrial fibrillation; CAD, coronary artery disease; MASLD, metabolic dysfunction–associated steatotic liver disease; MI, myocardial infarction.

## Data Availability

The data that support the findings of this study are not publicly available due to ethical and legal restrictions related to patient confidentiality but can be made available by the corresponding author upon reasonable request.

## Supplementary Materials

The following supporting information can be downloaded at https://www.mdpi.com/article/10.3390/medsci14020167/s1, Figure S1: PRISMA-style flow diagram illustrating patient selection for the retrospective HFpEF cohort; Figure S2: Cumulative incidence curves illustrating the association between HFpEF clusters and (a) in-hospital mortality and (b) composite short-term outcome; Table S1: Baseline characteristics in patients hospitalized for Heart Failure With Preserved Ejection Fraction; Table S2: Cause-specific hazards from the cause-specific Cox proportional hazard model.
