# Supplementary material for: Influence of Phenotypes on Short-Term Outcomes in Hospitalized Heart Failure with Preserved Ejection Fraction—Insights from a North-Eastern Romanian Cohort
Source: Med Sci (Basel). 2026 Mar 27;14(2):167. doi: 10.3390/medsci14020167 (PMC13108072; doi:10.3390/medsci14020167)
Supplement: Supplementary file 1 [file medsci-14-00167-s001.zip › medsci-4169923-supplementary.pdf]

Supplementary Material

Figure S1. PRISMA - style flow diagram illustrating patient selection for the retrospective HFpEF cohort.

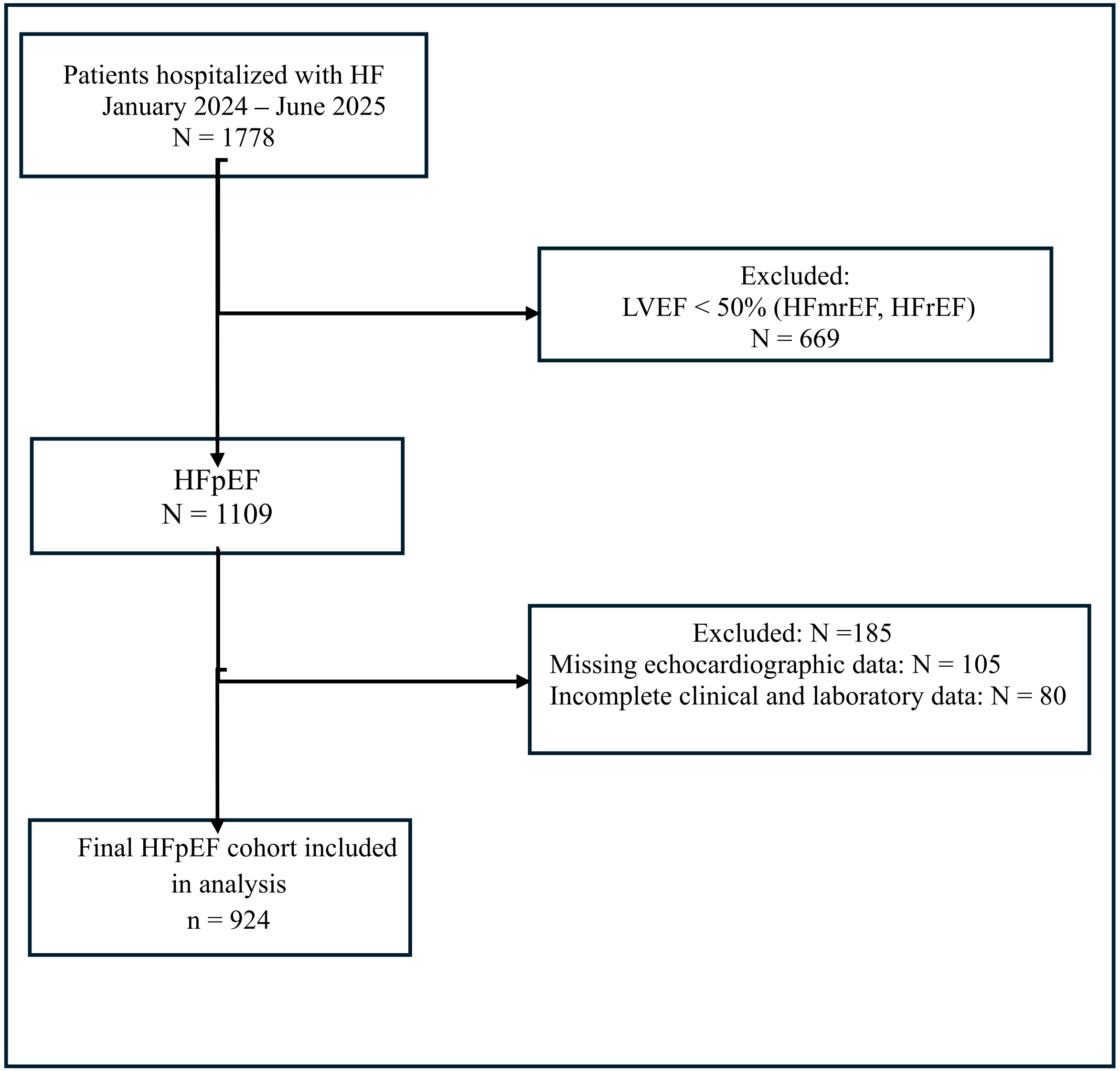

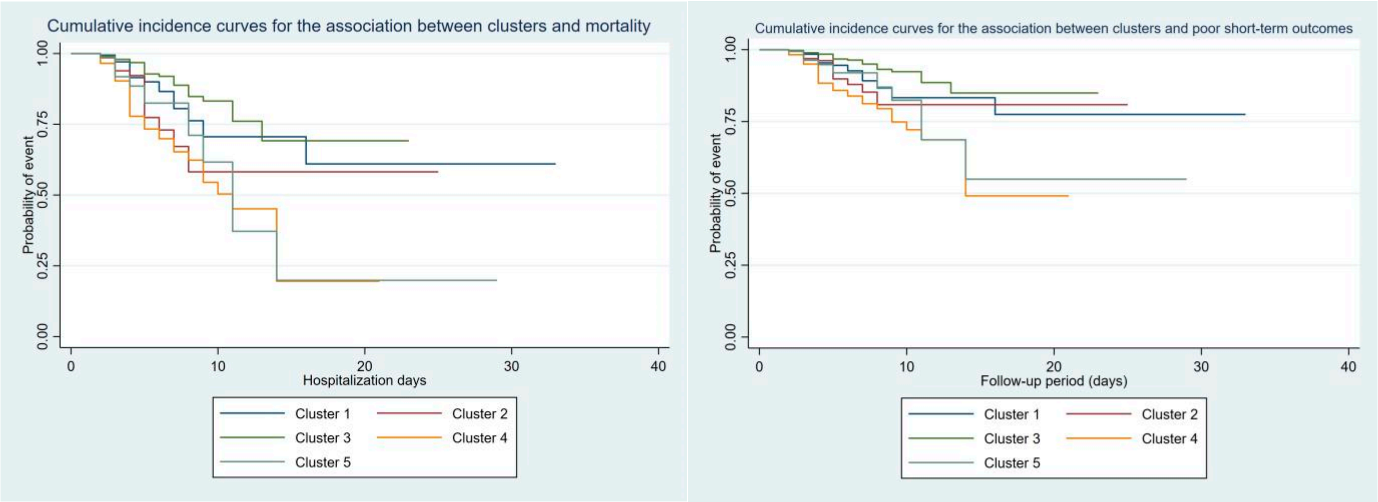

a **b**

Figure S2. Cumulative incidence curves illustrating the association between HFpEF clusters and (a) in-hospital mortality and (b) composite short-term outcome. Figure created using data from SPSS

Table S1. Baseline characteristics in patients hospitalized for Heart Failure With Preserved Ejection Fraction

**Variables**	**Value**	**% missing**
**Patients, n**	924	0
**Demographics**		
Age (years), median [IQR]	74 [67-81.0]	0
Female sex, n (%)	553 (59.8)	0

**HF measurements**		
H2FPEF score	4 [3-5]	0
NYHA class I/II, n (%)	764 (82.7)	0
NYHA class III/IV, n (%)	160 (17.3)	
**Signs and symptoms, n (%)**		
Dyspnea	514 (55.6)	0
Edema	363 (39.3)	0
Palpitations	123 (13.3)	0
Angina	174 (18.8)	0
**Clinical measurements**		
SBP (mmHg), median [IQR]	139 [125-154]	0
HR, bpm, median [IQR]	77 [68-89]	0
BMI (kg/m2), n (%)<br>< 25<br>≥ 25	 195 (21.1)<br>729 (78.4)	0
**Comorbidities, n (%)**		
Cardiovascular		
Atrial fibrillation	397 (43.0)	0
Hypertension	861 (93.2)	0

Valvular disease	635 (68.7)	0
ASCVD	597 (64.6)	0
Implantable devices*, n (%)	31 (3.4)	0
Noncardiovascular		
COPD	63 (6.8)	0
Sleep apnea	47 (5.1)	0
DM/ Prediabetes	414 (44.8)	0
Obesity	408 (44.1)	0
CKD / eGFR (mL/min/1.73m2), n (%)<br>≥60<br>30–60<br><30	 630 (68.2)<br>237 (25.6)<br>46 (5.0)	 0
Malignant cancer	73 (7.9%)	0
**Laboratory investigations**		
Biomarkers		
NT-proBNP (pg/mL), median [IQR]	666 [190-2570]	0
hscTnI (ng/L), median [IQR]	5.25 [1.85-15.05]	0
Hemoleucogram		
Hb (g/dl)	13 [11.5-14.4]	0
WBC	7300 [5955-9250]	0
Creatinine	0.87 [0.71-1.10]	0
Blood glucose	105 [93-126]	0

EKG<br>Normal EKG<br>Ischemia or myocardial infarction<br>Tachyarrhythmias or bradyarrhythmias<br>AF or AFL<br>Pacemaker rithm EKG	 476 (51.5)<br>77 (8.3)<br>55 (6.0)<br>297 (31.1)<br>17 (1.8)	0
Echocardiography		
LVEF, 50-55 %	632 (68.4)	0
LVEF, 56-64 %	239 (25.9)	0
LVEF, ≥ 65 %	50 (5.4)	0
**Medication upon dicharge, n (%)**		
Diuretic	573 (62.0)	0
ACE inhibitor/<br>ARB/ARNI	697 (75.4)	0
Beta-blocker	708 (76.6)	0
CCB	460 (49.8)	0
MRA	321 (34.7)	0
Antiaritmics	85 (9.2)	0
Statin	804 (87.0)	0
Antiplatelet	247 (26.7)	0

Anticoagulant	417 (45.1)	0
iSGLT2	408 (44.2)	0
Nitrates	125 (13.5)	0
**Outcomes n (%)**		
In-hospital death	22 (2.4)	0
30-day rehospitalization for HF	126 (13.6)	0

* Implantable cardioverter-defibrillator, cardiac resynchronization therapy or pacemaker
Data are presented as mean±SD, median (25th to 75th percentile) for continuous measures, and n (%) for categorical measures
IQR, interquartile range; NYHA, New York Heart Association; NT-proBNP, N-terminal pro-B-type natriuretic peptide; SBP, systolic blood pressure; HR, heart rate; BMI, body mass index; PAD, Peripheral Arterial Disease; ASCVD, Atherosclerotic Cardiovascular Disease; COPD, chronic obstructive pulmonary disease; CKD, chronic kidney disease; eGFR, estimated glomerular filtration rate; hscTnI, high-sensitivity cardiac Troponin I; Hb, Hemoglobin; AF, Atrial Fibrillation; AFL, Atrial Flutter; LVEF, left ventricular ejection fraction; ACE indicates angiotensin-converting enzyme; ARB, angiotensin receptor blocker; ARNI, Angiotensin-converting enzyme inhibitors; CCB, dihydropyridine calcium-channel blocker; iSGLT2, Sodium-Glucose Co-Transporter 2 inhibitor

Table S2. Cause-specific hazards from the cause-specific Cox proportional hazard model

Cluster	In-hospital mortality	Composite short-term outcome	HF re-hospitalisation
**1**	Reference	Reference	Reference
**2**	0.8 (95% CI 0.2 – 2.5)	1.0 (95% CI 0.7 – 1.6)	1.0 (95% CI 0.7 – 1.6)
**3**	1.2 (95% CI 0.1 – 11.4)	0.8 (95% CI 0.2 – 3.9)	2.2 (95% CI 0.8 – 5.9)
**4**	1.0 (95% CI 0.1 – 19.0)	2.1 (95% CI 0.7 – 6.4)	2.1 (95% CI 0.7 – 6.1)
**5**	1.3 (95% CI 0.3 – 5.6)	1.1 (95% CI 0.6 – 2.0)	1.1 (95% CI 0.6 – 2.0)
